# *Glaesserella parasuis ClpX* participates in stress tolerance and contributes to bacterial pathogenicity

**DOI:** 10.1128/spectrum.00497-25

**Published:** 2025-08-12

**Authors:** Manman Xu, Youqiao Fang, Bohong Li, Wenbin Wei, Zesong Wang, Qi Cao, Chen Tan, Ruicheng Yang, Huanchun Chen, Xiangru Wang

**Affiliations:** 1 National Key Laboratory of Agricultural Microbiology, College of Veterinary Medicine, Huazhong Agricultural University 627716, Wuhan, Hubei, China; 2 Key Laboratory of Preventive Veterinary Medicine in Hubei Province, The Cooperative Innovation Center for Sustainable Pig Production, Wuhan, Hubei, China; 3 Engineering Research Center of Animal Biopharmaceuticals, The Ministry of Education of the People’s Republic of China (MOE) 12543, Wuhan, Hubei, China; 4 Frontiers Science Center for Animal Breeding and Sustainable Production, Wuhan, Hubei, China; Michigan State University, East Lansing, Michigan, USA

**Keywords:** *Glaesserella parasuis*, *ClpX*, stress tolerance, pathogenicity

## Abstract

The *ClpX* gene in bacteria, which plays a key role in adaptation to various environmental stresses, is associated with pathogenicity. However, its role in conferring external environmental stress resistance in *Glaesserella parasuis* remains unclear. In this study, we used the clinically isolated serotype 5 CF7066 strain (wild type) to investigate the effects of *ClpX* on the external environmental stress response of *G. parasuis* by constructing *ClpX* mutant (*Δ-ClpX*) and complemented strains. Our data indicated that the viable bacterial count of *Δ-ClpX* was significantly lower than that of the wild type under high temperature, osmotic pressure, and oxidative stress conditions. Moreover, the *Δ-ClpX* strain exhibited extreme sensitivity to complement-mediated killing compared with the wild-type strain, whereas its serum-resistance ability was largely restored after *ClpX* gene complementation. Additionally, *ClpX* knockdown significantly attenuated the pathogenicity of the wild-type strain, and gene complementation largely recovered its phenotype in mouse and pig models. Taken together, our findings reveal *ClpX* as a novel virulence gene in *G. parasuis*, contributing to a deeper understanding of the pathogenic mechanisms employed by this bacterium.

**IMPORTANCE:**

The *ClpX* gene in bacteria is crucial for adapting to environmental stresses and is linked to pathogenicity. However, its role in conferring stress resistance in *Glaesserella parasuis* is unclear. This study investigates the impacts of *ClpX* on *G. parasuis*’ stress response by creating a *ClpX* mutant (*Δ-ClpX*) and complemented strain. The *Δ-ClpX* had reduced viability under heat, osmotic pressure, and oxidative stress conditions. It was also more sensitive to complement-mediated killing but regained serum resistance with *ClpX* complementation. *ClpX* knockdown reduced the wild-type strain’s pathogenicity in mice and pigs, which was largely restored by gene complementation. Thus, *ClpX* is identified as a novel virulence factor in *G. parasuis*, enhancing our understanding of its pathogenic mechanisms.

## INTRODUCTION

*Glaesserella parasuis*, a gram-negative bacterium belonging to the *Pasteurellaceae* family, is commonly found in commercial pig farms as an early colonizer of piglets (1). This non-motile, NAD-dependent bacterium represents a significant pathogen impacting the global pork industry (2). It frequently induces acute systemic inflammation in weaned and nursery pigs, leading to mortality due to meningitis, arthritis, and pneumonia (3). Traditionally, *G. parasuis* strains have been categorized into 15 serovars, some of which are associated with virulence (4), although their virulence factors remain incompletely defined. Both virulent and non-virulent strains can coexist in the upper respiratory tract of pigs (5). Under conditions of host stress or immune compromise, these bacteria may breach the respiratory barrier, enter the bloodstream, and cause systemic diseases characterized by severe fibrinous inflammation and respiratory syndromes, collectively termed Glässer’s disease (6, 7). Secondary infections often occur in conjunction with other pathogens, such as porcine reproductive and respiratory syndrome virus or other members of the *Pasteurella* genus, exacerbating morbidity and mortality rates and complicating control efforts (8).

Protein quality control systems have recently emerged as critical regulators of the life cycles in both prokaryotes and eukaryotes (9). These systems efficiently degrade denatured and misfolded proteins, thereby maintaining intracellular homeostasis (10). Extensive research on model organisms has revealed that protein quality control not only sustains cellular stability but also modulates gene expression under specific conditions (11). The orderly degradation of proteins within cells is predominantly governed by ATP-dependent proteolytic machinery, with AAA+ proteases playing a pivotal role in bacterial protein quality control (12). Notably, ClpX, a well-characterized AAA+ protease, is implicated in various essential functions such as stress responses and energy metabolism in microorganisms (13). Furthermore, evidence suggests that protein degradation participates in bacterial regulatory processes; the activation or inhibition of certain regulatory proteins influences bacterial activities, including growth, differentiation, sporulation, and stress response, as well as virulence (14).

Bacteria are highly sensitive to various environmental factors, including temperature fluctuations, nutrient availability, oxidative stress, and other conditions during natural growth (15). These adverse conditions can result in the accumulation of misfolded proteins, which can be detrimental to bacterial survival. Over the course of long-term evolution, bacteria have developed adaptive mechanisms that alter their physiological states and metabolic pathways to cope with environmental changes and maintain internal stability (16). Consequently, to ensure protein homeostasis under both normal metabolic conditions and stress stimuli, bacteria possess a sophisticated protein quality control system comprizing proteases and chaperones. This system facilitates the degradation of misfolded or unnecessary proteins, thereby recycling amino acids and energy for the synthesis of new functional proteins (17). For instance, bacteria can produce an extracellular matrix to form biofilms, enhancing their resistance to environmental stress in their natural habitats (18).

Previous studies have demonstrated that the proteases ClpX and ClpP are integral to *Streptococcus suis* stress tolerance and significantly contribute to its virulence (19). ClpX has been shown to play a crucial role in *Corynebacterium*’s resistance to environmental stress and energy metabolism (20). In *G. parasuis*, the protein degradation system is primarily encoded by ClpX and ClpP (21), with ClpP influencing the bacterium’s resistance to macrolides. However, the function of ClpX in *G. parasuis* remains underexplored. This study aimed to characterize the ATP-dependent Clp protease ClpX in the *G. parasuis* CF7066 strain, focusing on its role in stress tolerance and pathogenicity. Our findings enhance the understanding of bacterial infection mechanisms and provide insights into the pathogenic processes of *G. parasuis*.

## RESULTS

### Verification of the ClpX deletion mutant and its complementation strain

**Fig 1:**
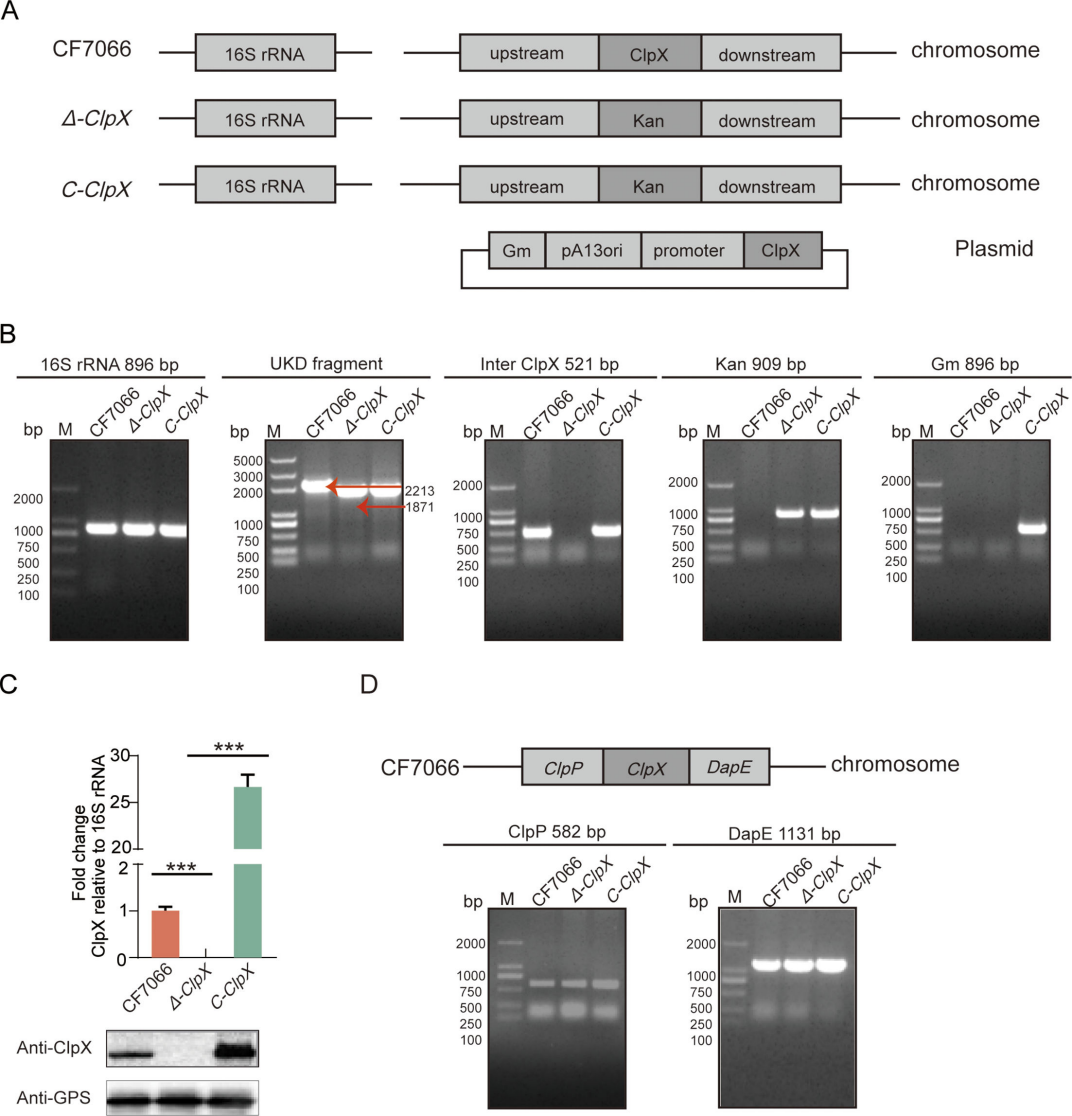
PCR identification of the *Glaesserella parasuis ClpX* deletion mutant (*Δ-ClpX*) and complementation (C-*ClpX*) strains. (**A**) Schematic representation of the construction process for the *ClpX* deletion strain. The upstream and downstream homologous arms were fused with the kanamycin resistance (Kan^r^) cassette and subsequently cloned into the pK18-mobsacB plasmid. The deletion strain was generated through natural transformation. For complementation, the *ClpX* gene was inserted into the pSHK3-Gm plasmid and introduced into the deletion strain *via* electroporation to restore *ClpX* expression. (**B**) PCR analysis confirmed the correct integration of each fragment in the respective strains. (**C**) Real-time PCR validation of *ClpX* mRNA transcription levels in the three experimental strains, along with Western blot analysis demonstrating ClpX protein expression. ***P < 0.001. (**D**) PCR verification of the expression levels of genes upstream and downstream of *ClpX*.

The *G. parasuis ClpX* deletion mutant strain (*Δ-ClpX*) was constructed from the wild-type strain CF7066 through natural transformation, as detailed in “Construction and validation of *ClpX* deletion mutant and its complementation strain” section. The complemented strain, C-*ClpX*, was generated by introducing the entire *ClpX* operon into the shuttle vector pSHK3-Gm. The *ClpX* mutant was created using homologous recombination (Fig. 1A). PCR analysis of the wild-type, *Δ-ClpX*, and C-*ClpX* strains (Fig. 1B) revealed an 896 bp 16S rRNA fragment in all three strains. Amplification of the UKD fragment, which contains upstream and downstream homologous arms with an intermediate region, using primers P1/P4, produced a slightly larger band in the *Δ-ClpX* strain compared to the wild-type strain due to the insertion of the kanamycin resistance cassette, which is slightly longer than the native *ClpX* sequence. Primer pairs *ClpX*-F/R, Kan-F/R, and Gm-F/R were used to detect the presence of *ClpX* fragments, kanamycin resistance cassettes, and gentamicin resistance genes, respectively. Real-time quantitative reverse transcription PCR (qPCR) confirmed the absence of the *ClpX* fragment in the *Δ-ClpX* strain but its presence in the wild-type CF7066 strain. Western blotting demonstrated that ClpX protein was detectable in lysates of both the wild-type CF7066 and the complemented C-*ClpX* strains, but not in the *Δ-ClpX* strain (Fig. 1C). PCR amplification of the upstream *ClpP* and downstream *DapE* genes showed expected band patterns (Fig. 1D). Collectively, these results confirm the successful knockout of the *ClpX* gene and the appropriate generation of the complemented strain.

### *G. parasuis ClpX* knockdown attenuates bacterial serum resistance and biofilm formation

**Fig 2:**
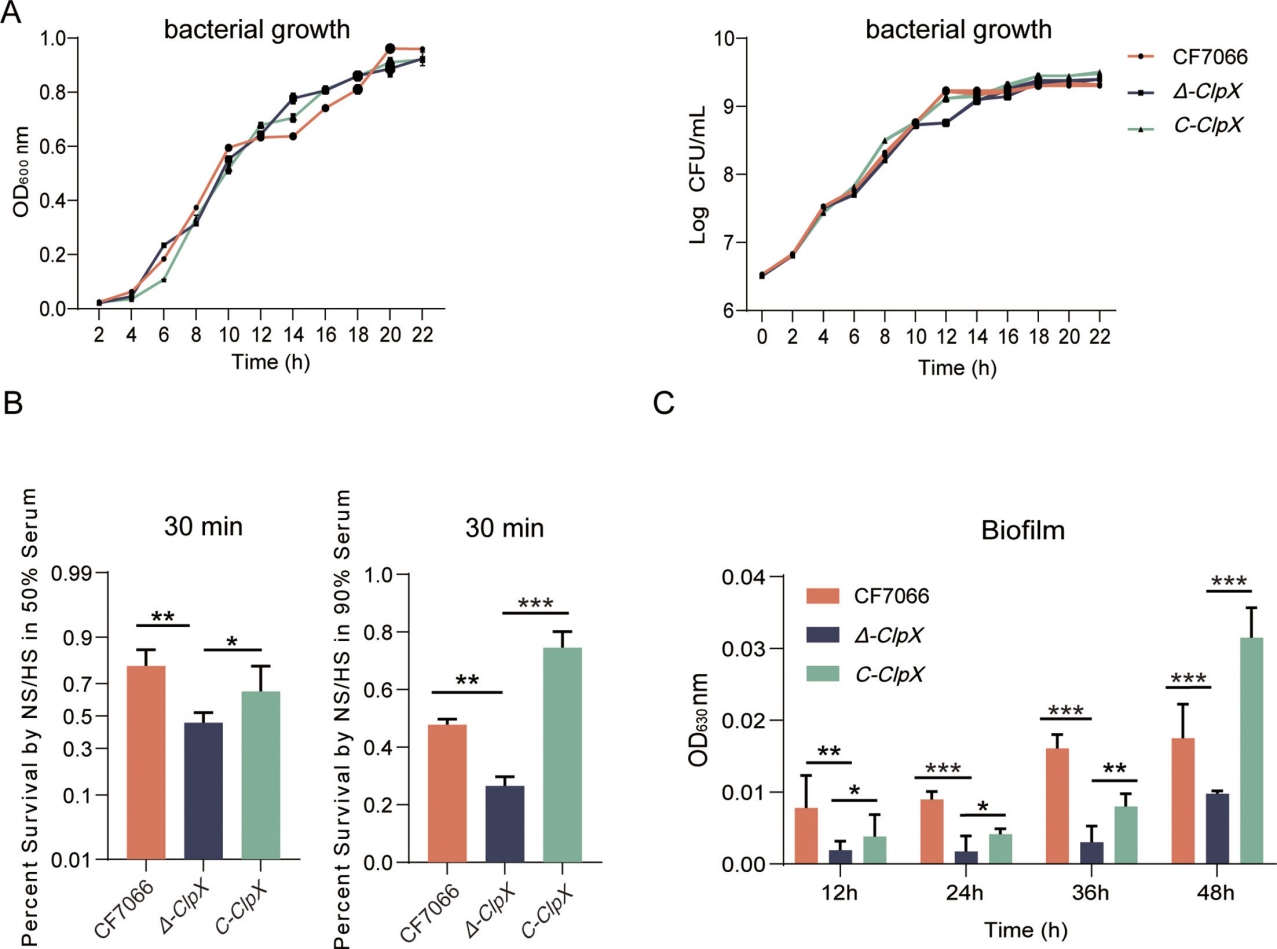
Comparative analysis of growth, serum resistance, and biofilm formation ability among wild-type CF7066, *ClpX* mutant (*Δ-ClpX*), and C-*ClpX* complementation strains. (**A**) All strains were cultured under identical conditions for 22 h. The OD_600_ and viable bacterial counts were measured at 2-h intervals. Data points represent the mean values from three independent replicates, with error bars indicating standard deviations. (**B**) Survival rates of wild-type, *Δ-ClpX*, and C-*ClpX* strains in response to 50% and 90% porcine serum. Percent survival was calculated as the ratio of bacterial colonies surviving in normal serum (NS) relative to those surviving in heat-inactivated serum (HS). (**C**) Biofilm formation capacity of wild-type, *Δ-ClpX*, and C-*ClpX* strains, expressed as absorbance values at 630 nm. Statistical significance: ***P < 0.001; **P < 0.01; and *P < 0.05.

Initially, we examined the impact of *ClpX* knockdown on the growth characteristics of *G. parasuis*. In comparison with the wild-type CF7066 strain, the loss of the *ClpX* gene did not result in any significant growth impairments (Fig. 2A). Growth curves for the wild-type CF7066, *Δ-ClpX* mutant, and complemented C-*ClpX* strains indicated similar growth patterns at 37°C, as evidenced by comparable viable cell counts and OD_600_ values. These findings suggest that *ClpX* does not play a critical role in the growth of *G. parasuis*.

Systemic infection by *G. parasuis* necessitates the effective invasion of different tissues and cells by circulating bacteria. A prerequisite for this capability is the pathogen’s survival in the host’s bloodstream, which hinges on its resistance to complement-mediated killing (22). In this study, the serum-resistance experiment demonstrated that the CF7066 strain exhibited superior survivability following incubation with 50% or 90% porcine serum for 30 min (Fig. 2B). In contrast, the *Δ-ClpX* mutant displayed increased sensitivity to complement-mediated killing, resulting in a significantly lower survival rate compared to the wild-type strain. Complementation of the *Δ-ClpX* mutant restored the serum-resistant phenotype. These findings underscore the critical role of *ClpX* in enabling *G. parasuis* to withstand complement-mediated killing.

 Various serovars of *G. parasuis* have been shown to form biofilms *in vitro*, which play a crucial role during infections (23). To examine the differences in biofilm formation among CF7066, *Δ-ClpX*, and C-*ClpX* under identical culture conditions, quantitative analysis was performed using a microtiter plate assay at multiple time points (Fig. 2C). Notably, after 12 h of incubation, a significant difference in biofilm formation was observed between the CF7066 and *Δ-ClpX* strains, whereas no significant difference was detected between C-*ClpX* and CF7066. These findings suggest that the complementation strain can restore biofilm formation to levels comparable to those of the wild-type strain, indicating that *ClpX* may enhance biofilm formation. Collectively, these results demonstrate that *ClpX* plays an important role in facilitating *G. parasuis* biofilm formation, particularly under adverse external conditions.

### *ClpX* is required for bacterial resistance to oxidative, osmotic, and temperature stresses

**Fig 3:**
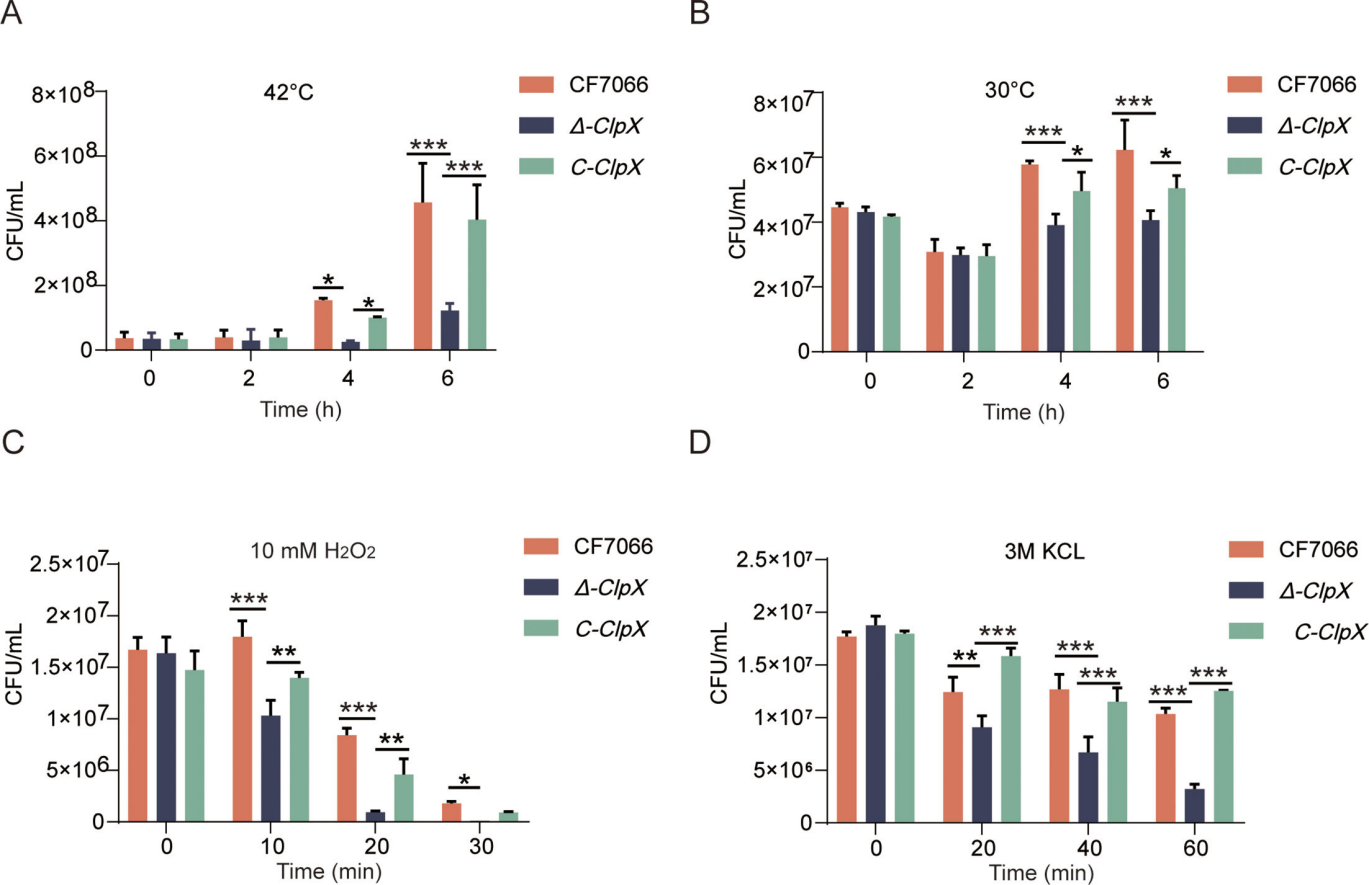
Evaluation of *ClpX* in bacterial tolerance to temperature, oxidative, and osmotic stress conditions. Bacterial cells from wild-type CF7066, *Δ-ClpX* mutant, and C-*ClpX* complementation strains, harvested at mid-exponential phase, were subjected to viability assays under various stress conditions: (**A**) heat stress at elevated temperatures, (**B**) cold stress at reduced temperatures, (**C**) oxidative stress induced by 10 mM H_2_O_2_, and (**D**) osmotic stress induced by 3M KCl. Statistical significance was determined as follows: ***P < 0.001; **P < 0.01; and *P < 0.05.

Given that *Δ-ClpX* exhibited distinct phenotypes during serum resistance and biofilm production compared with wild-type strain, we further investigated the role of *ClpX* in the infection process of *G. parasuis* by evaluating the ability of test strains to withstand various culture conditions. These conditions included temperature shock, oxidative stress, and osmotic pressure, all of which are encountered by the host during infection. To simulate the effects of pathogenic bacteria infecting the host and resisting inflammation and elevated temperatures, we measured the growth of three test strains at high (42°C) and low (30°C) temperatures (Fig. 3A and B). After 6 h of continuous culture, the growth under thermal stress of *Δ-ClpX* was significantly less compared to the complemented strain, while the wild-type strain maintained the largest number of viable bacteria, whether it was cultured at 30°C or 42°C. Viable cell counts indicated a reduction in bacterial numbers for the *ClpX* mutant strain under both high- and low-temperature conditions.

In the oxidative tolerance assay, we sequentially assessed the viable cell counts of the three test strains in the presence of 10 mM H_2_O_2_. Our enumeration data revealed that the *Δ-ClpX* strain exhibited significantly reduced viability when exposed to H_2_O_2_ after a 10-min incubation at 37°C compared to both the wild-type and complemented strains. Notably, almost all *Δ-ClpX* mutant cells were eliminated following a 30-min incubation (Fig. 3C). These findings underscore the critical role of *ClpX* in *G. parasuis*’s antioxidative stress response.

Given that *ClpX* has been shown to contribute to stress tolerance under oxidative and high-temperature conditions, we investigated its potential involvement in osmotic stress. To this end, bacterial cells were incubated in trypticase soy broth (TSB) supplemented with 3M KCl. Following a 1-h incubation under conditions identical to those used in the oxidative and temperature tolerance assays, it was observed that the viability of *Δ-ClpX* mutant bacteria was significantly diminished compared to both the wild-type and complemented strains (Fig. 3D). These results support the hypothesis that *ClpX* may play a role in the osmotic stress response. Collectively, these findings indicate that the *ClpX* gene is crucial for bacterial adaptation to diverse environmental challenges.

### *ClpX* plays crucial functions in facilitating the adherence and invasion of *G. parasuis* to host cells

**Fig 4:**
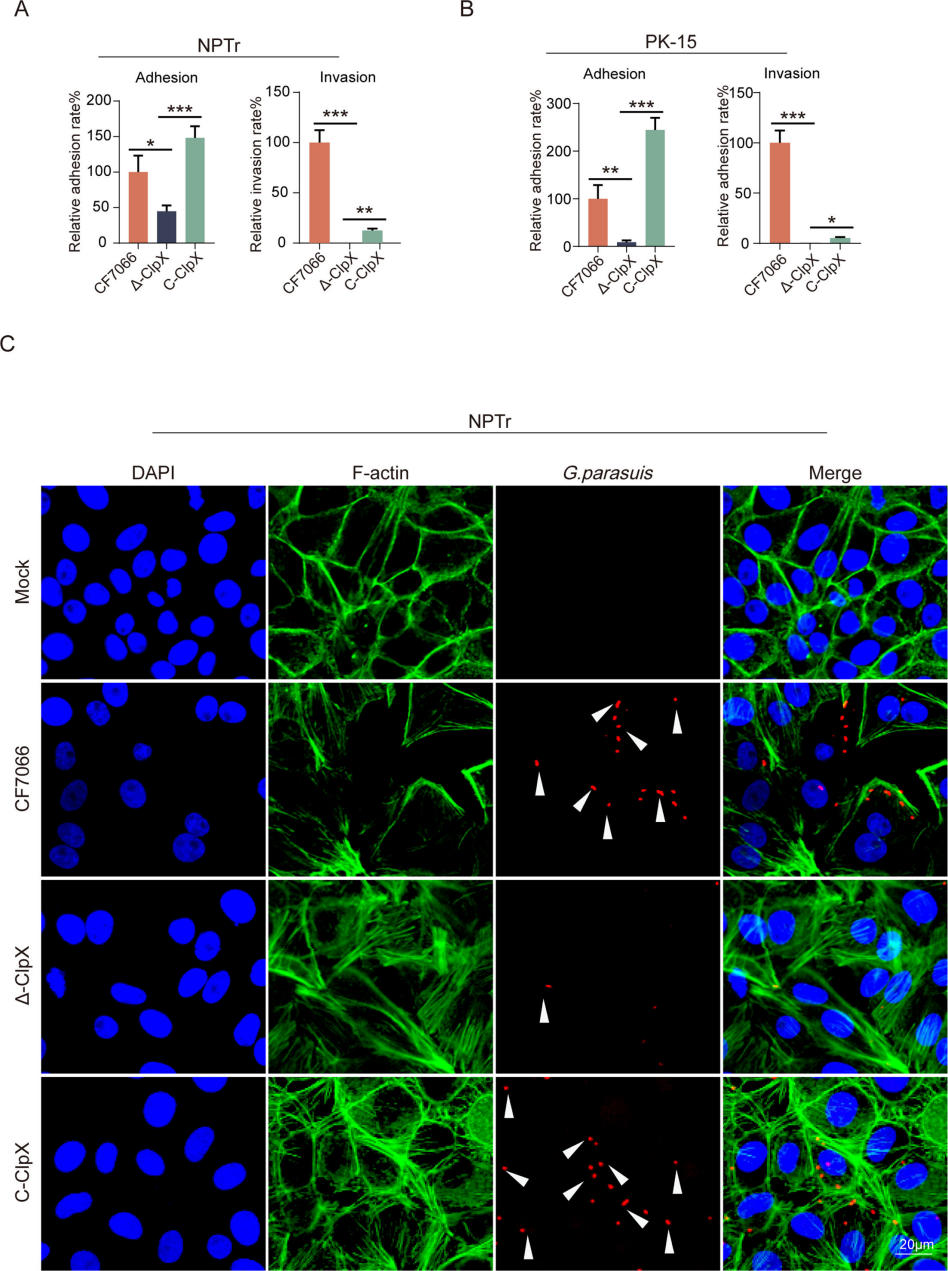
Effects of *ClpX* knockdown on adhesion and invasion of *G. parasuis* in porcine cells. The adhesion and invasion rates of (**A**) NPTr cells and (**B**) PK-15 cells by the wild-type strain CF7066, the *Δ-ClpX* mutant, and the C-*ClpX* complemented strain were quantified. Data are presented as the ratio of bacteria adhered to or invaded into cells relative to the initial inoculum added to each well of a 24-well plate. (**C**) Confocal fluorescence micrographs of cells following adhesion and invasion assays. Uninfected cells (Mock) served as controls. F-actin was stained with Actin-tracker Green, nuclei with DAPI, and *G. parasuis* with Cy3 red. Statistical significance: ***P < 0.001; **P < 0.01; and *P < 0.05.

During the entire infection and pathogenesis process, the adhesion and invasion of pathogenic microorganisms to host cells play a critical role, particularly in establishing initial infection and adhering to and invading respiratory epithelial cells (24). To further investigate the effects of *G. parasuis ClpX* on host cell interactions, we incubated newborn pig trachea (NPTr) and porcine kidney (PK-15) cell lines with planktonic wild-type, mutant, and complemented strains to compare their adherence and invasion capabilities. The *Δ-ClpX* mutant exhibited significantly reduced adherence and invasion in both cell lines compared to the wild-type and C-*ClpX* strains. Notably, the C*-ClpX* strain demonstrated significantly higher adhesion abilities than the wild-type strain in both cell lines (Fig. 4A and B). However, the restoration of ClpX did not significantly enhance the invasion ability of the mutant strain, indicating potential differences in the mechanisms by which ClpX promotes bacterial adhesion versus invasion. To validate our findings, we conducted confocal laser microscopy on post-infected NPTr cell samples. Red fluorescence was used to label adherent *G. parasuis*, which revealed that the *Δ-ClpX* mutant had significantly fewer red fluorescent cells compared to the wild-type and complemented strains (Fig. 4C).

### *ClpX* is implicated in the virulence of *G. parasuis*

**Fig 5:**
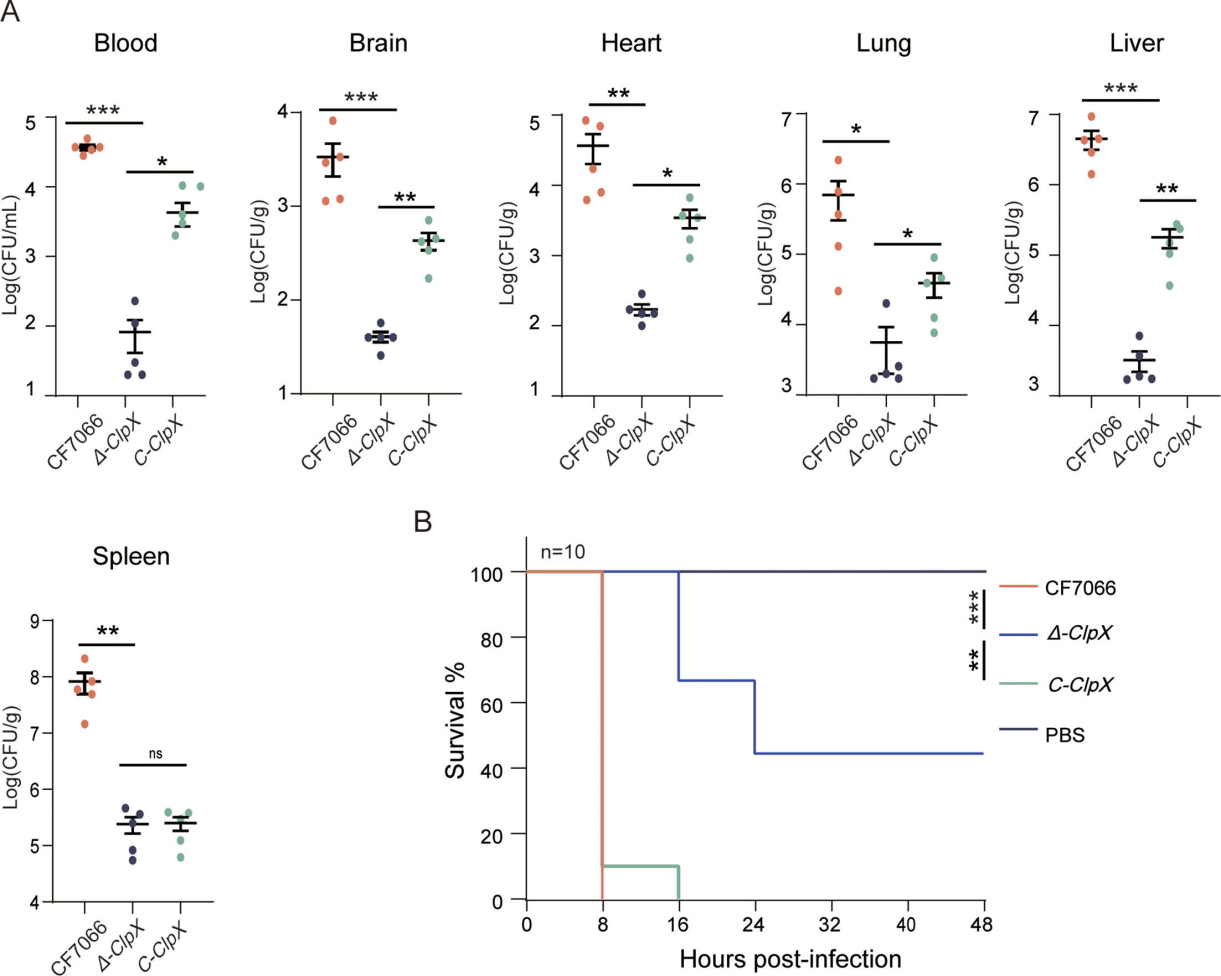
Effect of *ClpX* knockdown on systemic infection *in vivo*. (**A**) Colonization levels of the wild-type CF7066, *Δ-ClpX* mutant, and C-*ClpX* complementation strains in various mouse tissues. Groups of five female BALB/c mice were intraperitoneally inoculated with 5 × 10^8^ CFU/mouse. Bacterial loads in blood, brain, heart, lung, liver, and spleen were quantified at 8 h post-infection. (**B**) Survival rates of mice challenged with the wild-type, *Δ-ClpX*, and C-*ClpX* strains. Groups of 10 female BALB/c mice were intraperitoneally inoculated with 1.5 × 10^8^ CFU/mouse. Survival data were analyzed using the log-rank (Mantel-Cox) test. ***P < 0.001; **P < 0.01; and *P < 0.05.

Subsequently, we aimed to demonstrate that the *Δ-ClpX* mutant exhibits diminished resistance to external stress relative to both the wild-type and complemented strains. To investigate the role of *ClpX* during *in vivo* colonization by *G. parasuis*, mice were infected. Bacterial isolates were obtained from blood, brain, heart, lung, liver, and spleen at 8 h post-infection across three groups of mice (Fig. 5A). The number of recovered *Δ-ClpX* bacteria was significantly lower compared to the wild-type strain. The bacterial burden in the organs of mice challenged with the complemented strains was comparable to that observed in the wild-type strain.

 To evaluate the survival curves of mice, three experimental groups, each comprizing 10 specific pathogen-free (SPF) BALB/c female mice aged 4 weeks, were intraperitoneally inoculated with 1.5 × 10^9^ CFU of the bacterial strains of interest. A control group of 10 SPF BALB/c mice received an equivalent volume of sterile phosphate-buffered saline (PBS) via intraperitoneal injection. Survival rates were monitored at 8 h intervals for 7 days post-infection. All mice challenged with the wild-type strain exhibited clinical symptoms including respiratory distress, disheveled coats, unresponsiveness to stimuli, lethargy, and tremors within 6 h of infection, and all succumbed within 8 h. Mice exposed to the complemented strain died within 16 h. In contrast, the *Δ-ClpX* strain-infected group exhibited milder clinical symptoms and lower mortality (5/10) over the 1-week observation period. These findings are summarized in Fig. 5B. Collectively, these results indicate that the *Δ-ClpX* strain’s ability to infect and colonize was significantly compromised in the murine model, underscoring the importance of *ClpX* as a virulence factor in systemic *G. parasuis* infections.

### Depletion of *ClpX* in *G. parasuis* mitigated clinical manifestations in infected piglets

To further investigate the functional role of ClpX in the pathogenesis of *G. parasuis*, a controlled challenge experiment was conducted using twelve 25-day-old weaned piglets. Clinical findings indicated that no piglets within the control group (PBS) displayed any clinical manifestations. In contrast, piglets infected with CF7066 developed symptoms characteristic of Glässer’s disease, which included elevated body temperature (>40.5°C), anorexia or reduced appetite, dyspnea, cyanosis of the skin, joint swelling, and lameness. In advanced stages, ruffled hair, weight loss, lethargy, and mortality were observed. Necropsies performed on deceased piglets revealed severe serous and fibrinous exudates, with all fatalities occurring within 5 days post-infection. Similar clinical manifestations were noted in the C-*ClpX* group; however, only one piglet died in the *Δ-ClpX* group.

**Fig 6:**
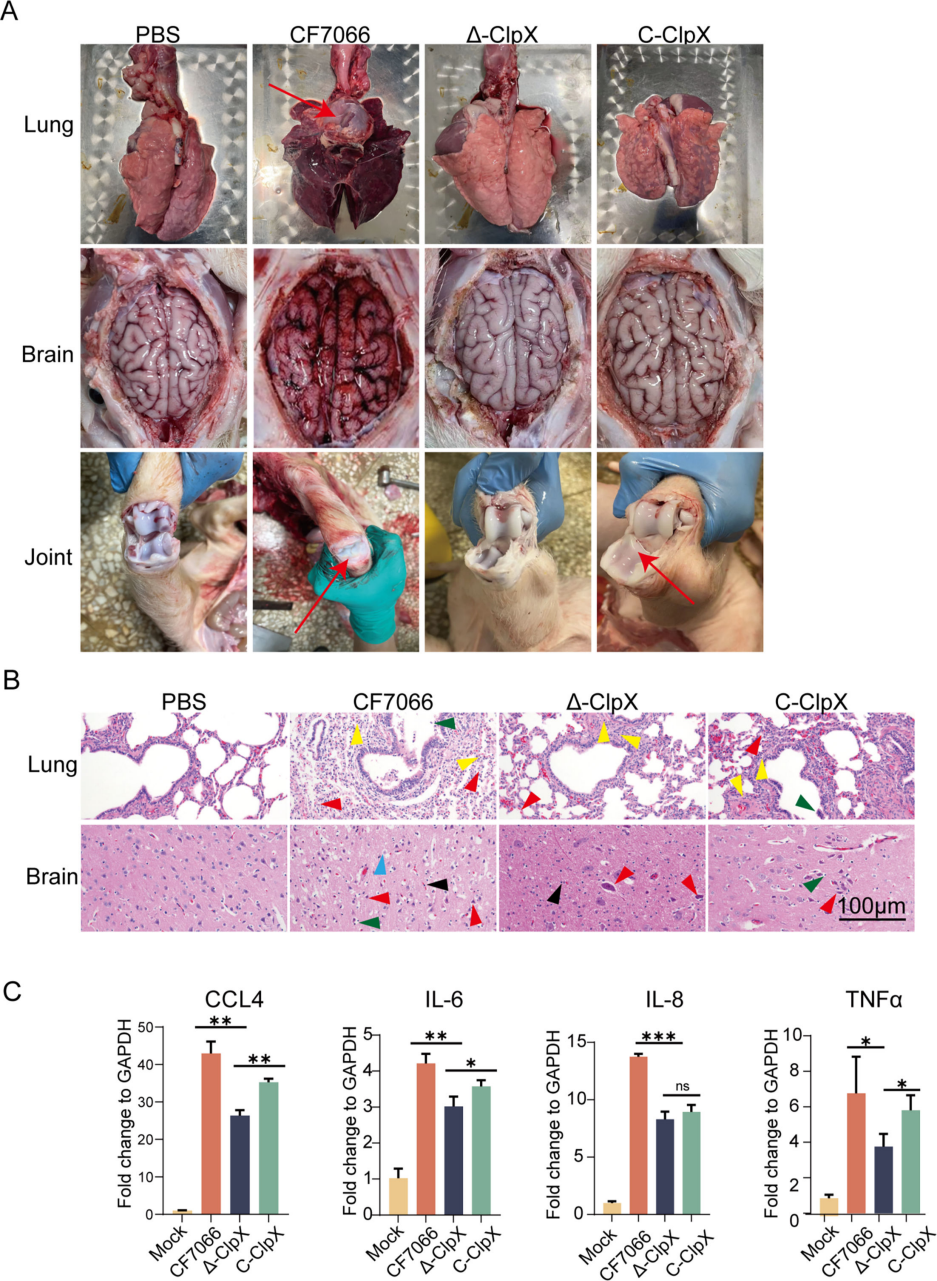
Inflammatory lesions and histopathological examination in piglets. (**A**) Gross anatomical observations of the lungs, brains, and joints from four groups of piglets. Red arrows indicate fibrinous exudates. (**B**) Hematoxylin and eosin staining of lung and brain tissues in response to *G. parasuis* infection. Scale bar indicates 100 µm. (**C**) Transcription levels of inflammatory cytokines in 3D4/21 macrophages infected with the wild-type CF7066, *Δ-ClpX* mutant, and C-*ClpX* complementation strains (MOI = 100). Statistical significance: ***P < 0.001; **P < 0.01; and *P < 0.05.

The clinical anatomy is illustrated in Fig. 6A. Hematoxylin and eosin staining (Fig. 6B) demonstrated that infection with the wild-type strain caused the structure of lung tissue to be severely abnormal, including: (i) loss of distinct alveolar boundaries with marked hyperplasia of alveolar epithelial cells, (ii) alveolar collapse associated with thickened alveolar walls (red arrow), (iii) luminal accumulation of inflammatory cells in bronchioles (green arrow), and (iv) dense inflammatory cell infiltration within the alveolar septa (yellow arrow), whereas the *Δ-ClpX* mutant group exhibited relatively intact tissues with mild lesions. This trend of pathological changes was also observed in brain tissue: wild-type-infected brains exhibited profound neuropathological alterations, including: (i) cortical architectural disorganization with neuronal degeneration evidenced by cytoplasmic vacuolation and karyopyknotic nuclei (red arrow); (ii) reactive gliosis manifested as increased density of GFAP^+^ cells in the cerebral cortex (black arrow); (iii) satellitosis surrounding residual neurons (green arrow); and (iv) focal edema characterized by cellular swelling and cytoplasmic pallor (blue arrow). However, no pathological phenomenon of edema and necrosis was observed in the brain tissue of the *Δ-ClpX* strain-infected group.

To investigate the impact of *ClpX* on the inflammatory response elicited by *G. parasuis*, CF7066, *Δ-ClpX*, and C-*ClpX* strains were used to infect 3D4/21 cells for 24 h, followed by qPCR analysis of inflammatory cytokine mRNA levels (Fig. 6C). Post-infection, CF7066 significantly upregulated the transcriptional expression of inflammatory factors such as CCL4, IL-6, IL-8, and TNF-α, exhibiting the highest mRNA levels. Conversely, the *Δ-ClpX* strain showed a reduction in inflammatory factor expression, while the C-*ClpX* strain demonstrated improvements. These findings suggest that *ClpX* expression in *G. parasuis* enhances pathogenicity, leading to pronounced inflammatory damage to the lungs and brain through the promotion of inflammatory responses.

## DISCUSSION

The foundation of infectious diseases lies in the intricate interactions between pathogenic microorganisms and host cells. Following infection, a multitude of signal transduction pathways and gene expression profiles in both microorganisms and host cells undergo significant alterations (25). These changes activate a series of intracellular immune responses, leading to disruptions in biological processes such as cell proliferation, differentiation, inflammation, and apoptosis, ultimately resulting in disease development. When pathogens invade, they must withstand the host’s counterattack to establish systemic infection (26). Upon surviving this adverse environment, respiratory pathogens must re-adapt to the tracheal environment to proliferate and establish infection. Evidence suggests that protease-dependent protein turnover observed during recovery from host counterattacks not only effectively eliminates toxic protein aggregates but also facilitates protein synthesis by providing readily available amino acids during this critical transition from stress to non-stress conditions. This prepares respiratory bacteria for reinitiation after stress relief, necessitating rapid restoration of energy metabolism and the formation of building blocks for protein synthesis (27). The AAA+ protease, *ClpX*, plays a crucial role in stress tolerance and virulence across various bacterial species including *Mycobacteria*, *Streptococcus*, *Xanthomonas campestris*, *Streptococcus mutans*, *Bacillus subtilis*, *Yersinia enterocolitica,* and *Listeria monocytogenes* (28–30). Notably, *ClpX* exhibits up to 99% homology among *G. parasuis* strains. Interestingly, *G. parasuis* also demonstrates significant homology with ClpX proteins from other gram-negative bacteria (Fig. S1). As a symbiotic colonizer of the porcine upper respiratory tract, *G. parasuis* must have encountered diverse stress challenges prior to its successful survival and invasion *in vivo*. The specific role of *ClpX* in enabling *G. parasuis* to overcome these stresses and facilitate infection has not been previously elucidated, and the underlying mechanisms remain to be determined.

Complement-mediated bacterial killing is a key component of the humoral arm of innate immunity necessary to control systemic infection from mucosal sources. Encapsulated bacteria thwart the antibacterial effect of complement by preventing deposition of the membrane attack complex on bacterial membranes (31). Previous literature has shown that lipopolysaccharide is the major factor mediating complement resistance (32, 33). Our experiments revealed that the *ClpX* deletion mutant exhibited significantly lower survival rates in both 50% and 90% serum compared to wild-type and complemented strains (Fig. 2B). C3b and MAC are key players in the activation of complement-mediated killing. We speculate that whether ClpX, as a protease, degrades directly or indirectly, not to mention related molecules, is the focus of our next research direction.

Limited information is available about the role of ClpX in bacterial attachment. In *S. mutans*, the ClpX mutant strain revealed less biofilm mass in the presence of glucose, whereas the biofilm formation was enhanced when sucrose was provided (34). Our experiments revealed that the *ClpX* deletion mutant exhibited a reduction in biofilm formation capability. Their markedly different properties may suggest that those proteins associated with serum resistance and biofilm are substrates of ClpX.

 Bacteria possess diverse genetic elements that confer adaptive advantages under various environmental stresses (35). The expression of numerous bacterial proteins is regulated at the transcription initiation stage in response to varying environmental conditions. This study sought to evaluate whether ClpX proteins are critical factors enabling *G. parasuis* to withstand different environmental stresses. Previous research has demonstrated that ClpX deletion mutants in other bacterial species exhibit significantly reduced stress tolerance due to the absence of ATPase specificity factors that bind to Clp proteases (36, 37). Our results indicated that the *Δ-ClpX* strain exhibited heightened sensitivity to temperature shock, oxidative stress, and osmotic pressure compared to other tested strains (Fig. 3). Moreover, both the wild-type and C-*ClpX* strains displayed enhanced resilience relative to the *Δ-ClpX* mutant strain, consistent with findings from a previous study on *Staphylococcus aureus Δ-ClpX* strains. Therefore, it can be inferred that the ClpX protease plays a crucial role in the survival of pathogenic microorganisms under adverse conditions. Further investigation is required to elucidate the precise mechanism by which ClpX contributes to the stress response in *G. parasuis*. One potential explanation for the increased sensitivity of *Δ-ClpX* strains to these stresses may involve the proteolytic regulation of regulatory proteins targeted by *ClpXP* during the transcriptional control of downstream genes (38).

 The initial stage of respiratory pathogen infection involves the adherence to and invasion of host cells, thereby compromising the respiratory barrier and leading to systemic infection (39). Previous studies have identified this capability as a critical virulence factor (40). Our findings revealed that the *Δ-ClpX* strain exhibited a significant reduction in both adhesion and invasion compared to the CF7066 strain, supporting the observed decrease in virulence in the *Δ-ClpX* mutant (Fig. 4). Similar observations have been reported in *C. crenatum* (20). In addition, according to the results, ClpX is more involved in the bacterial adhesion process, and the adhesion factor may act as a substrate for ClpX to enhance the degradation of the binding protein (41).

 The mechanism underlying virulence attenuation following *ClpX* knockdown in *G. parasuis* remains to be elucidated. During the critical stages of infection, *G. parasuis* can survive in the bloodstream after transmission via the respiratory tract (42). The reduced tolerance of the *Δ-ClpX* mutant to various environmental stresses may be a significant factor contributing to the attenuated virulence of *G. parasuis*, as these mutant cells are less likely to endure within the host environment (43). In a mouse infection model, mice challenged with the *Δ-ClpX* strain exhibited prolonged survival times compared to those challenged with the wild-type and complemented strains (Fig. 5). Additionally, the bacterial burden retrieved from mice infected with the *Δ-ClpX* strain was significantly lower in multiple organs, including the blood, brain, heart, lung, liver, and spleen, relative to that observed in mice infected with the wild-type strain. Similar findings have also been observed in *S. aureus* (37). Collectively, it is reasonable to infer that *ClpX* plays an important role in influencing the virulence of *G. parasuis*.

With a focus on elucidating the role of *ClpX in vivo*, we particularly evaluated the pathogenicity differences among these three strains of *G. parasuis* by intraperitoneally challenging piglets. Given that the lungs are the primary target organ for *G. parasuis* colonization (44), histopathological examinations were carried out and revealed that piglets infected with CF7066 exhibited severe lung damage and substantial inflammatory cell infiltration (Fig. 6), consistent with Wilkinson et al.’s *in vivo* studies (45). Taken together, this study underscores the critical role of *ClpX* in mediating virulence and stress tolerance in *G. parasuis*. These *in vitro* and *in vivo* findings significantly enhance our understanding of the Clp protease’s function in the pathogenesis of *G. parasuis*. Future research should aim to elucidate the precise mechanisms by which ClpX regulates bacterial stress tolerance, thereby contributing to the virulence of *G. parasuis*.

## MATERIALS AND METHODS

### Bacterial strains, plasmids, cells, and culture conditions

The bacterial strains and plasmids utilized in this study are listed in Table 1. The virulent *G. parasuis* strain CF7066 (wild type) served as the parent strain for gene deletion experiments. All relevant strains were cultured on trypticase soy agar (TSA) or TSB (Difco, Detroit, MI, USA), supplemented with 0.001% (wt/vol) NAD (Sigma-Aldrich, St. Louis, MO, USA) and 5% (vol/vol) heat-inactivated bovine serum (Zhejiang Tianhang Biotechnology Co., Ltd., 23022-8615) at 37°C. When necessary, the growth medium was further supplemented with kanamycin (50 µg/mL) or gentamicin (20 µg/mL). *Escherichia coli* DH5α was grown in Luria–Bertani (LB) medium at 37°C. Antibody of ClpX and *G. parasuis* was previously obtained and preserved in our laboratory. The anti-*G*. *parasuis* antibody was previously collected from the infected mice by our laboratory and has been extensively used in our previous work, and anti-ClpX was prepared by immunizing the recombinant ClpX protein in mice.

**TABLE 1:** Bacterial strains and plasmids used in this study

Strains and plasmids	Characteristics	Source or reference
Wild Type	*G. parasuis* serotype 5**,** field isolate, CF7066	(2)
Δ-*ClpX*	*ClpX* mutant of CF7066, Kan^r^	This study
C*-ClpX*	Complementation strain of *ΔClpX::kan* containing pSHK3-C-*ClpX*, Kan^r^, Gm^r^	This study
pK18mobsacB	Suicide and narrow-broad-host vector, Kan^r^	(46)
pK18-Δ-*ClpX*::kan	A 2,213 bp overlap fragment containing Kan^r^, the upstream and downstream sequences of the *ClpX* gene in pK18mobsacB, Kan^r^	This study
pSHK_3_	*E. coli–G. parasuis* shuttle vector, Kan^r^	(47)
pSHK_3_-Gm	Kan^r^ replaced by Gm^r^ (534 bp) in pSHK_3_, Gm^r^	This study
pSHK_3_-C-*ClpX*	A fragment containing the 1,251 bp promoter and complete *ClpX* ORF in pSHK_3_-Gm, Gm^r^	This study

PK-15 (ATCC, CCL-33) and NPTr cell (48, 49) lines were maintained in Dulbecco’s modified Eagle’s medium (Invitrogen, Carlsbad, CA, USA) supplemented with 10% heat-inactivated fetal bovine serum (FBS) (SORFA, SX1101) in a humidified incubator at 37°C with 5% CO_2_ until monolayer confluence was reached. 3D4/21 (ATCC, CRL-2843) cells were cultured in RPMI 1640 (Gibco, 31800022) medium supplemented with 10% FBS, 10 mM l-glutamine, 10 mM sodium pyruvate, 10 mM nonessential amino acids and 10 mM HEPES buffer (PH = 7.2–7.4) (Solarbio, H1095).

### Construction and validation of *ClpX* deletion mutant and its complementation strain

The primers utilized in this study are detailed in Table 2. Genomic DNA was extracted from *G. parasuis* CF7066 and served as the template for PCR amplification. The upstream and downstream fragments flanking *ClpX* were amplified from the CF7066 genome using the primer pairs P1/P2 and P3/P4, respectively. Additionally, a kanamycin-resistant (kan^r^) cassette was amplified from the pSHK3 vector. These three PCR fragments (upstream, kan^r^, and downstream) were subsequently integrated via overlap PCR using primers P1 and P4. The resulting fusion segment was digested with EcoRI and BamHI and ligated into the similarly digested pK18mobsacB vector to construct the pK18mobSacB-*ClpX*-UKD plasmid. This plasmid was introduced into *G. parasuis* CF7066 through natural transformation, following a previously described protocol (50), with slight modifications. Specifically, 8 mM cAMP was added to the recipient bacterial suspension during logarithmic phase (OD_600_ of approximately 1.0). Subsequently, 1 µg of donor DNA plasmid was added to the bacterial mixture, which was then spread onto TSA plates containing 50 mg/mL kanamycin at 37°C. After incubation, all natural transformants on the plates were identified by PCR using primer pairs P1/P4. For complementation studies, the *ClpX* operon, including its natural promoter and terminator regions, was cloned into *E. coli–G. parasuis* shuttle vector pSHK3-Gm, generating the complementation plasmid pSHK3-C-*ClpX*. This plasmid was introduced into the *Δ-ClpX* mutant *via* electroporation.

**TABLE 2:** Primers used in this study^*a*^

Primers	Sequence (5′−3′)	*T*_m_
16sRNA-F/R	GGCTTCGTCACCCTCTGT GTGATGAGGAAGGGTGGTGT	59°C
*ClpX*-upstream P1/P2	**GGAATTC**AACGCTTGTAATGGCGGTGTTGAAGAT (EcoRI) TTTTTATCTTGTGCAATGTATTCACTATCTCTTTGTTACAACG	51°C
*ClpX*-downstream P3/P4	ATCAGAATTGGTTAATTGCTTAATTGATATTCTCCCCGTC **CGGGATCC**ACAAGCGGTGATCCCAGGCGAGCTTTA (BamHI)	51°C
*ClpX*-F/R	**GGAATTC**ATGGCAAATTTTGAGAAAGAACC (EcoRI) **CCGCTCGAG**CTCCACCTTCGGCTTTTCACTT (XhoI)	58°C
Kan-F/R	CATTGCACAAGATAAAAATA CAATTAACCAATTCTGATTA	50°C
Gm-F/R	ATGTTACGCAGCAGCAACG TTAGGTGGCGGTACTTGGGTC	59°C
Inter-*ClpX*-F/R	CGTGGCATTATTTTTATTGATGAAATCGAC GTGAATTTTAATTCCACACCTTCCATTTTG	58°C
DapE-F/R	ATGCAAAATCAAATTATCTTTCTTAGTCG CTAATTTGGTATAACCGAACACAAAAT	55°C
ClpP-F/R	CTATCTCTTTGTTACAACGTCATCAA ATGGCTGTAATCCCTATGGT	55°C
q16sRNA-F/R	TGAAGTCGGAATCGCTAGTA CCTACGGTTACCTTGTTACG	60°C
qCCL-4 F/R	CTCCTGCTGCTATACACTTAC CAGTTCAGTTCCAAGTCATCCAT	60°C
qIL-6 F/R	AATCCAGACAAAGCCACCAC TCCACTCGTTCTGTGACTGC	60°C
qIL-8 F/R	AGTTTTCCTGCTTTCTGCAGCT TGGCATCGAAGTTCTGCACT	60°C
qTNF-α F/R	GCTCTTCTGCCTACTGCACTTC GTCCCTCGGCTTTGACATT	60°C

^*a*^ Boldface indicates the restriction enzyme site, which has been shown following the sequence.

### Growth characteristics determination

To compare the growth rates of the wild-type CF7066, *Δ-ClpX* mutant, and complemented C-*ClpX* strains, overnight cultures were grown in 5 mL TSB medium and subsequently diluted to an OD_600_ of 1.0 in the same medium. These cultures were then inoculated into fresh medium at a 1:1,000 dilution and incubated at 37°C with orbital agitation at 180 rpm for 22 h. OD_600_ measurements were taken hourly using an Eppendorf BioSpectrometer (Eppendorf, Hamburg, Germany). Colony-forming units (CFUs) were determined by plating serial dilutions of culture aliquots every 2 h. All experiments were conducted independently in triplicate.

### Serum resistance assay

Porcine serum was collected from healthy piglets that were confirmed to be free of Glässer’s disease and devoid of *G. parasuis* antibodies. The serum underwent filter sterilization (0.22 µm) and was subsequently stored at −80°C until required for use. Certain aliquots were subjected to heat treatment at 56°C for 30 min to inactivate complement components. The serum resistance assay was conducted according to a previously established protocol (51), with minor modifications. For the 50% serum bactericidal assay, 100 µL of bacterial cultures at 1.0 × 10^6^ CFU/mL was combined with 100 µL of either fresh or heat-treated sera. The mixtures were incubated at 37°C for 30 min with gentle agitation (130 rpm/min), followed by serial 10-fold dilutions and plating on TSA medium. Incubation then proceeded at 37°C for 24 h. For the 90% serum bactericidal assay, 20 µL of bacterial cultures at 5.0 × 10^6^ CFU/mL was mixed with 180 µL of fresh or heat-treated sera, after which all subsequent steps mirrored those of the 50% serum bactericidal assay. Results are expressed as percent survival, calculated by dividing the number of bacteria treated with normal serum (NS) by those treated with heat-inactivated serum (HS). Each strain was assayed in triplicate.

### Biofilm formation assay

Biofilm formation assays were conducted in 96-well microtiter plates (Thermo Fisher, Waltham, MA, USA) following the methodology outlined in a previous study (52). The OD_600_ of overnight cultures was standardized to 0.6 using the same protocol as described in “*ClpX* plays crucial functions in facilitating the adherence and invasion of *G. parasuis* to host cells” section for growth analysis. Each well received 20 µL of inoculum added to 180 µL of TSB and incubated at 37°C for various durations (12, 24, 36, and 48 h). Following incubation, the supernatant was aspirated, and each well was washed three times with 200 µL of sterile PBS to eliminate loosely adherent cells. The remaining bacterial biofilms were fixed with methanol for 30 min. After air-drying, the wells were stained with a 1% crystal violet solution for 10 min at room temperature. Excess stain was removed, and the wells were rinsed until the effluent water appeared clear. Plates were then dried in a 37°C incubator for 30 min. Biofilms were subsequently solubilized with 33% (vol/vol) glacial acetic acid, and the OD_630_ was measured using an Eppendorf BioSpectrometer. Uninoculated wells served as negative controls. All experiments were performed in triplicate and repeated six times.

### Survival assays of *G. parasuis* under temperature, oxidative, and osmotic stress conditions

To evaluate the role of the *ClpX* gene in response to various risk factors, wild-type, *Δ-ClpX,* and C-*ClpX* strains were subjected to multiple stress conditions, including temperature shifts, oxidative stress (H_2_O_2_), and osmotic stress (KCl). Overnight cultures of *G. parasuis* were adjusted to an OD_600_ of 1.0. Bacterial cultures were subsequently centrifuged and washed two times with PBS prior to each experiment. For the temperature shock assay, the cells were incubated at 42°C or 30°C for 6 h in a water bath. In the oxidative stress tolerance assay, cell suspensions were exposed to 10 mM H_2_O_2_ at 37°C for 30 min. For the osmotic stress tolerance assay, the hyperosmotic challenge system was established by inoculating 100 µL of a standardized bacterial suspension into 10 mL of filter-sterilized 3M potassium chloride (KCl) solution, which was prepared using deionized water (Milli-Q Advantage A10, resistivity 18.2 MΩ·cm at 25°C). After the respective incubation periods, the cells were serially diluted in PBS, and CFUs were determined by plate counting. All assays were conducted independently in triplicate on three separate occasions.

### Adhesion and invasion assays

Adhesion assays were conducted using PK-15 and NPTr cells according to a previously established protocol (53, 54). Specifically, 5.0 × 10^5^ cells were seeded into each well of 24-well tissue culture plates and maintained at 37°C in a humidified incubator with 5% CO_2_ for 24 h. Subsequently, the monolayers were washed three times with PBS and subjected to serum starvation overnight in serum-free medium. Bacterial cultures were prepared by growing bacteria overnight in 5 mL TSB at 37°C with agitation. The bacterial cells were then harvested, washed, resuspended in fresh cell culture medium, and inoculated onto the confluent cell monolayers at a concentration of 2.0 × 10^7^ CFU/well. To enhance contact between *G. parasuis* and the cell surface, the plates were centrifuged at 800 × *g* for 10 min (55). For the adhesion assay, the plates were incubated at 37°C for up to 2 h to facilitate bacterial adhesion. Following this incubation period, non-specifically adhered bacteria were removed by thorough washing with PBS. The cells were then treated with 200 µL of 0.05% trypsin/0.03% EDTA solution for 10 min at 37°C. After treatment, the cell suspensions containing adherent bacteria were collected from the bottom of each well, serially diluted 10-fold, and plated on TSA agar.

For the invasion assay, cell culture preparation, bacterial infection, and bacterial enumeration were conducted following the procedures outlined for the bacterial adherence assay. Thereafter, the cells were washed three times to eliminate unbound bacteria and subsequently incubated in media containing 100 U/mL penicillin and 10 µg/mL streptomycin sulfate for 1 h to eliminate extracellular bacteria. Following this, the cells were washed and lysed according to the previously described methods for the bacterial adherence assay. All experiments were performed in triplicate, and the entire procedure was replicated three times.

### Immunofluorescence

For the cell immunofluorescence assay, NPTr cells were seeded and cultured in glass-bottomed dishes (Ø35 mm) for 12 h. Subsequently, the cells were infected with three bacterial strains at an MOI of 100 for 2 h. Following this incubation period, the culture medium was aspirated. Immunofluorescence staining was conducted according to the manufacturer’s protocols provided with the staining kits. Specifically, after washing the cells three times with PBS, they were permeabilized using 1% Triton X-100 in PBS. Non-specific binding sites were blocked, and antibody incubations were performed as follows: the cells were fixed with 4% paraformaldehyde and blocked with PBS containing 5% BSA at 37°C for 2 h. Primary antibody (anti-*G*. *parasuis*) was then applied and incubated overnight at 4°C. After five washes with PBS, secondary antibodies were added and incubated for 1 h. *G. parasuis* was labeled with Cy3, while F-actin was stained with actin-tracker green. The samples were examined using a ZEISS LSM 880 Meta confocal laser scanning microscope (Carl Zeiss, Jena, Germany).

### Animal infection assays

Animal experiments were conducted in strict accordance with the guidelines outlined in the China Regulations for the Administration of Experimental Animals (1988) and were approved by the Institutional Animal Care and Use Committee of Huazhong Agricultural University (HZAUMO-2022-0182; Wuhan, China). A mouse model was utilized to evaluate the colonization and invasion capabilities of various bacterial strains in systemic organs, as previously described (56, 57). In this study, five 4-week-old SPF BALB/c female mice per test group were intraperitoneally injected with 5.0 × 10^8^ CFU/mouse of each bacterial strain. Upon the onset of clinical symptoms such as tachypnea and a disheveled coat, mice were humanely euthanized, and tissues (blood, brain, heart, lung, liver, and spleen) were collected 12 h post-infection. Samples from different organs were weighed, homogenized in PBS, and plated on TSA at 10-fold serial dilutions to determine the presence of colonizing bacteria. Colony counts were recorded as CFU/mL for blood samples and CFU/g for organ samples. For survival assays, mice were randomly assigned to four groups of 10 and intraperitoneally injected with 1.5 × 10^9^ CFU/mouse of the respective bacterial strains. Clinical symptoms were monitored daily for 7 days post-infection, and survival data were recorded accordingly. Kaplan-Meier survival curve analyzes were performed using GraphPad Prism v10.0 (GraphPad Software, La Jolla, CA, USA), and the significance of differences between groups was assessed using the log-rank (Mantel-Cox) test.

For *in vivo* pathological evaluation using a piglet model, twelve 25-day-old piglets were randomly assigned to four groups: wild-type, *Δ-ClpX*, C-*ClpX,* and negative control (PBS). Each group received an intraperitoneal injection of the respective bacterial strain at a concentration of 2.1 × 10^10^ CFU per piglet. Piglets exhibiting severe illness or nearing death were euthanized, as were surviving piglets 14 days post-infection. Lung and brain tissues were subsequently collected for histopathological analysis.

### Statistical analysis

All data were derived from a minimum of three independent experiments, each conducted in triplicate, and are presented as the mean ± standard deviation (SD). Statistical analysis and graphical representation were performed using GraphPad Prism software version 10.0. Statistical significance was defined as *P* < 0.05 (*), while extremely significant results were indicated by *P* < 0.01 (**) or *P* < 0.001 (***), respectively.

## SUPPLEMENTAL MATERIAL

Fig. S1 spectrum.00497-25-s0001.docx Multisequence alignment of the *G. parasuis* ClpX protein sequence with orthologs of various other bacteria.

ASM does not own the copyrights to Supplemental Material that may be linked to, or accessed through, an article. The authors have granted ASM a non-exclusive, world-wide license to publish the Supplemental Material files. Please contact the corresponding author directly for reuse.
