# Supplementary material for: Glaesserella parasuis ClpX participates in stress tolerance and contributes to bacterial pathogenicity
Source: Microbiol Spectr. 2025 Aug 12;13(9):e00497-25. doi: 10.1128/spectrum.00497-25 (PMC12403881; doi:10.1128/spectrum.00497-25)
Supplement: Fig. S1 — Multisequence alignment of the G. parasuis ClpX protein sequence with orthologs of various other bacteria. [file spectrum.00497-25-s0001.docx]

**Supplementary figure**

**Figure S1**

**
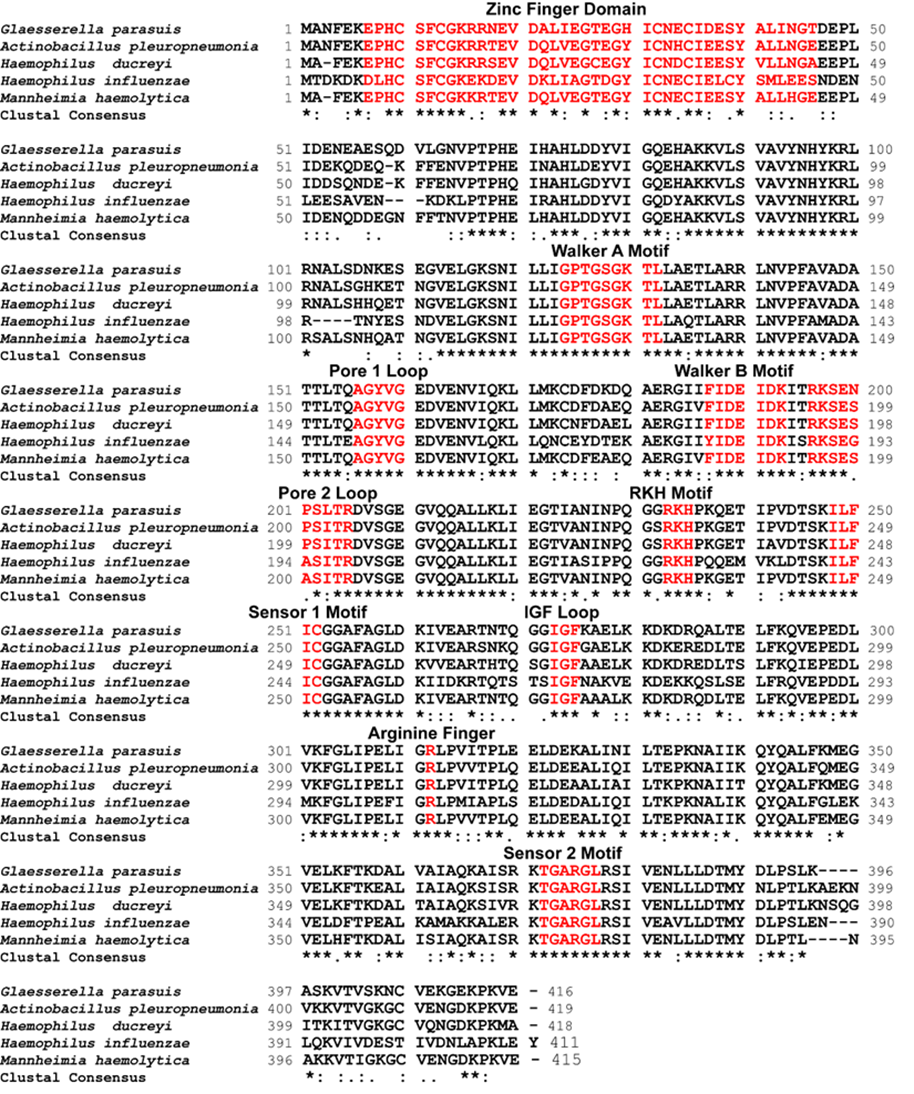
**

**Figure S1.** Multi-sequence alignment of the *G. parasuis* ClpX protein sequence with orthologs of various other bacteria. Red letters indicate different domains of the ClpX protein. DNAman software was used for multi-sequence alignment with the default settings.
